# Coordination of reach-to-grasp in physical and haptic-free virtual environments

**DOI:** 10.1186/s12984-019-0525-9

**Published:** 2019-06-27

**Authors:** Mariusz P. Furmanek, Luis F. Schettino, Mathew Yarossi, Sofia Kirkman, Sergei V. Adamovich, Eugene Tunik

**Affiliations:** 1 Department of Physical Therapy, Movement, and Rehabilitation Science, Northeastern University, 360 Huntington Ave., Boston, MA 02115 USA; 2 Department of Human Motor Behavior, the Jerzy Kukuczka Academy of Physical Education in Katowice, 72A Mikolowska St, 40-065 Katowice, Poland; 3 Psychology Department, Lafayette College, Easton, PA 18042 USA; 4 Department of Electrical and Computer Engineering, Northeastern University, 360 Huntington Ave., Boston, MA 02115 USA; 5 Department of Biomedical Engineering, NJIT, 323 Dr. Martin Luther King Jr. Boulevard, Newark, NJ 07102 USA; 6 Department of Rehabilitation and Movement Science, Rutgers University, 65 Bergen St, Newark, NJ 07107 USA; 7 Department of Bioengineering, Northeastern University, 805 Columbus Ave., Boston, MA 02120 USA

**Keywords:** Virtual reality, Kinematics, Prehension, Reach-to-grasp phases, Collision detection

## Abstract

**Background:**

Virtual reality (VR) offers unprecedented opportunity as a scientific tool to study visuomotor interactions, training, and rehabilitation applications. However, it remains unclear if haptic-free hand-object interactions in a virtual environment (VE) may differ from those performed in the physical environment (PE). We therefore sought to establish if the coordination structure between the transport and grasp components remain similar whether a reach-to-grasp movement is performed in PE and VE.

**Method:**

Reach-to-grasp kinematics were examined in 13 healthy right-handed young adults. Subjects were instructed to reach-to-grasp-to-lift three differently sized rectangular objects located at three different distances from the starting position. Object size and location were matched between the two environments. Contact with the virtual objects was based on a custom collision detection algorithm. Differences between the environments were evaluated by comparing movement kinematics of the transport and grasp components.

**Results:**

Correlation coefficients, and the slope of the regression lines, between the reach and grasp components were similar for the two environments. Likewise, the kinematic profiles of the transport velocity and grasp aperture were strongly correlated across the two environments. A rmANOVA further identified some similarities and differences in the movement kinematics between the two environments - most prominently that the closure phase of reach-to-grasp movement was prolonged when movements were performed in VE.

**Conclusions:**

Reach-to-grasp movement patterns performed in a VE showed both similarities and specific differences compared to those performed in PE. Additionally, we demonstrate a novel approach for parsing the reach-to-grasp movement into three phases- initiation, shaping, closure- based on established kinematic variables, and demonstrate that the differences in performance between the environments are attributed to the closure phase. We discuss this in the context of how collision detection parameters may modify hand-object interactions in VE. Our study shows that haptic-free VE may be a useful platform to study reach-to-grasp movements, with potential implications for haptic-free VR in neurorehabilitation.

**Electronic supplementary material:**

The online version of this article (10.1186/s12984-019-0525-9) contains supplementary material, which is available to authorized users.

## Background

Widespread accessibility to highly specialized and advanced digital technology has made virtual reality (VR) more and more common in daily life. While VR was originally developed as an enhancement for video games and entertainment, more recent applications of virtual environments (VE) are used in a number of rapidly growing fields including virtual assembly [1], medical training [2] and rehabilitation [3–6]. Each of these applications requires the user to manually interact with virtual objects within the immersive computer-generated environment. Depending on the particular goal, the user may need to reach and grasp to a diverse set of objects in order to manipulate their position or state.

Such reach-to-grasp movements are a standard of VE based upper-extremity (UE) neurorehabilitation [7]. Though devices exist to provide haptic feedback of object properties, these often-expensive devices [8] have numerous limitations. Namely, wearable haptic devices are heavy, leading to a slowing down of movement, and non-wearable haptic devices restrict natural motion in the environment, for review see [1, 9]. These factors, along with cost and availability, greatly limit the widespread application of VE for rehabilitation. In contrast, motion tracking has a broad spectrum of commercially available products that support mobility at low cost [10], therefore making haptic-free VE (hf-VE) a more accessible option. Key to its use in rehabilitation, VE provides the opportunity for adaptive and progressive motor learning through perceptual modifications such as activity and workplace scaling, and error augmentation, resulting in greater dosage, intensity and engagement in training [11, 12]. While this capability makes VE a powerful technology for rehabilitation, it is still unclear to what extent movements produced in hf-VE resemble those produced in the physical environment (PE).

A comprehensive characterization of the kinematic differences between reach-to-grasp movements produced in the two environments will provide important information regarding the feasibility of hf-VE for UE rehabilitation. Therefore, in this investigation we sought to systematically describe the differences between environments by characterizing phase specific differences the reach and grasp components of reach-to-grasp.

To date, there have been few investigations of reach-to-grasp movements in VE. Magdalon and collaborators [13] reported that subjects instructed to reach and grasp three types of virtual and physical objects (a can, a screwdriver and a pen) reached significantly slower in VE, with a relative time to peak velocity (rTPV) around 10% earlier and a longer relative deceleration time. Though aperture scaling was preserved in VE, peak aperture (PA) was wider in VE when grasping the smaller objects (screwdriver and pen). In a follow-up experiment by the same group [14], several prehension measures were compared across VE and PE in individuals who have had stroke. They concluded that participants used similar movement strategies, and subjects were able to scale aperture to object size but with longer delays between time to peak transport velocity (TPV) and PA. It is important to note that all of the above studies comparing movement kinematics in PE versus VE provided some sort of haptic feedback. Given the dearth of evidence about how reach-to-grasp is coordinated in VE without haptics, we set out to compare performance in hf-VE and PE.

It is generally agreed that the transport and the grasp, though relatively independently controlled, also show evidence of temporal coordination [14–20]. Some of this evidence includes a TPA at 60–70% of transport time as well as a PV during the first half of the movement [16, 21]. Levin et al. [14] tested the effects of VE with haptic feedback on the kinematics of reach-to-grasp movements. In order to quantify the coordination of the reach and grasp, the authors expressed temporal coordination as the delay between the times of arm peak velocity and hand maximal aperture. Their results showed that there were longer delays between the landmarks in VE with respect to movements in PE, suggesting a possible loss of coordination. Given the tight relationship between the transport and grasp in natural movements, our second goal was to determine if the kinematics of the individual components, as well as the coordination between the components, is preserved when performing under visual feedback alone (haptic-free) reach-to-grasp movements in VE.

We hypothesized that wider grip apertures and longer movement times would be observed in hf-VE (hypothesis 1). Moreover, we also hypothesized that coordination between the transport and grasp components would be preserved across environments (hypothesis 2). To test these hypotheses, we asked healthy subjects to produce reach-to grasp movements to size- position-matched physical and virtual objects of different sizes (manipulating the grasp component) and distances (manipulating the transport component) in both PE and VE.

## Methods

### Participants

Thirteen healthy subjects, 2 females (age: 23.9 ± 6.8 years old, body mass: 74.7 ± 9.9 kg, height: 1.7 ± 0.13 m) free from neurological, muscular, or orthopedic conditions, took part in the study. Prior to data collection, all participants provided informed consent approved by the Northeastern University Institutional Review Board (NU IRB# 15–10-22) with the ethical standards of the Helsinki Declaration. All participants were right-handed based on their preferred hand for writing, eating and throwing [21].

### Apparatus

Kinematic data were recorded from small IRED markers attached to the tips of thumb and index finger, and wrist at the center of the line running between the ulnar and radial styloid process. Marker data were captured using a six-camera active infrared motion tracking system (PPT Studio N, WorldViz, CA, USA). A custom 3D immersive VE (UNITY ver. 5.6.1f1, 64 bits, Unity Technologies SF) was displayed to the subject via the Oculus HMD - head-mounted display (Rift DK2, VR, LLC.). A pair of IRED markers on the HMD were used to co-register head movements with the VE. The virtual environment was calibrated so that the objects were located at the same distance as in the physical environment. Kinematic data was tied to the framerate of the HMD which was set to 75 Hz.

**Fig. 1:**
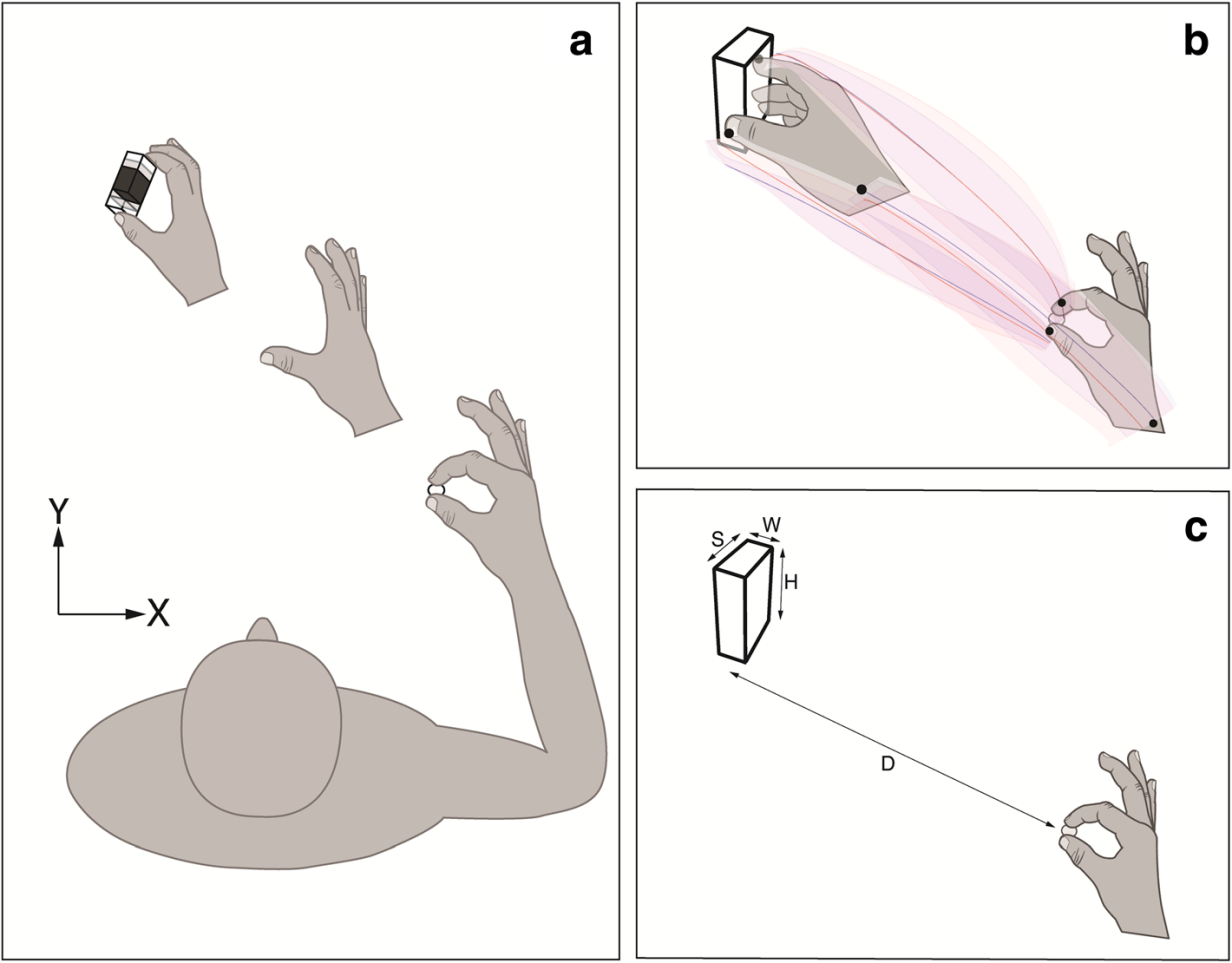
Schematic illustration of the experimental setup. **a** Position of the hand and example of one object distance (colors represent different object sizes: Large-white, Medium-gray, Small-black). **b** Initial and final position of the hand during reach-to-grasp movement with mean example trajectories (shade areas represents standard deviations, red-virtual environment, blue-physical environment). **c** Object parameters, D-distance of a hand to the object, S-object size, W-object width, H-object height

The objects used in the study (physical and virtual) were three different sized rectangular prisms with equal width (W) and height (H) of 2.5 cm and 8 cm, respectively. Sizes (S) of the graspable dimension were: Large - 7.2 cm, Medium - 5.4 cm, Small - 3.6 cm (see Fig. 1). During the experiment, the objects were placed in three different positions along the proximal to distal axis measured from the start switch to the center of an object (D): Near - 24 cm, Middle - 30 cm, Far - 36 cm). All objects were rotated along their vertical axis (Z) to 65 degrees measured from horizontal (X) axis to make them easier to grasp without excessive wrist extension (see Fig. 1). The physical objects were 3D printed using PLA thermoplastic filaments, and weighed 30, 44, and 59 g, for Small, Medium and Large, respectively.

## Procedure

Subjects sat comfortably in front of a table. They placed their dominant (right) hand on the table in a comfortable pinch position lightly holding a wooden peg which maintained a starting distance between thumb and index of 1.5 cm to prevent interference between the infrared markers. The position of the peg was 24 cm to the right, and 12 cm in front, of the body midline. The thumb was placed on the start switch next to the peg. Each trial began with an auditory signal, cuing the subject to reach, grasp, and lift the virtual or physical object (see Fig. 1). Subjects were instructed to reach naturally toward the object with the dominant upper limb, without leaning their trunk forward, at their preferred speed keeping their hand relatively parallel to the table to reduce vertical movements. The subjects’ view of their fingertip position in the VE condition was projected as two green spheres (0.8 cm, diameter) rendered at the 3D position of the fingertip IRED markers. To grasp the object, subjects placed the fingertip spheres of the thumb and index finger (pincer grip) approximately on the middle of each object’s lateral surface (see Fig. 1, panel b). Contact with the virtual object was controlled by a collision detection algorithm which colored the virtual object red when the fingertip spheres reached the location of the object (to make virtual objects easy to grasp the total collision error margin was set to 1.2 cm). As the object was lifted from the table, the subject kept it in the vertical position before putting the object back down (in VR, the object was held above the table until it disappeared) and returning their hand to the starting position. In PE, subjects completed the reach-to-grasp task in a dark room, seeing only the glow-in-the-dark object and the illuminated IRED markers on their fingertips (see Additional file 1: Video 1). Overhead lights were turned on between trials to prevent dark adaptation. Therefore, visual information remained similar across the environments.


**Additional file 1: Video 1.** (AVI 2110 kb)


Before data collection, subjects were familiarized with the setup and procedure, particularly to the VE environment. Familiarization consisted of 135 trials in VE (15 trials × 3 object sizes and × 3 distances). Five second ‘rest’ periods were provided after every 15 trials.

The experiment consisted of 216 trials (108 trials in the PE block, 108 trials in the VE block). Three seconds were allowed for each trial. After every 12 trials, a 5-s ‘rest’ period was provided. A 5-min ‘rest’ was provided between blocks.

### Analysis

Data were analyzed offline using custom Matlab routines (The Mathworks, Natick, MA). Trials were cropped from movement onset (start switch) to moment offset (contact of both thumb and index finger with the object). In the physical environment, offset was defined as the moment when aperture stopped decreasing. In VR, offset was defined as the timestamp when the virtual object was successfully grasped (thumb and index finger markers met the collision detection criteria). Kinematic data were lowpass filtered at 6 Hz with a 4th order Butterworth filter.

Based on the markers attached to fingertips (thumb, index), the following kinematics were calculated. Grasping components: movement time (MT) as the time between movement onset and offset, grip aperture as the 2D (horizontal plane) distance in time between thumb and index markers at each sample, peak aperture (PA) (maximum value of grip aperture), and time to peak aperture (TPA). Size normalized aperture at each sample was calculated by first subtracting the aperture at movement onset, −then normalizing this value to the aperture at movement offset (object contact). The rationale for this normalization was two-fold. First, markers were attached to the dorsum of the digits (on the nail) in order to not create a barrier between the finger pads and the object that would interfere with grasping. In the VE, the collision detection algorithm was dependent on the position of these markers. This created a discrepancy between PE and VE in the final grip aperture (at the time of grasp) roughly equal to the width of the finger from pad to nail. The described normalization accounts for this discrepancy. Second, normalization in this way permitted the direct comparison of aperture profiles and features, such as the size normalized peak aperture (snPA), between different size objects. To better understand motor planning, we also analyzed digit positions along the vertical object at movement offset (object grasp). Specifically, we calculated the vertical distance between the thumb and index finger. This measure is analogous to the COP difference that has been commonly calculated in studies of digit force planning [22, 23], though obviously lacks information about forces on the object.

Based on the wrist marker, the following transport components were calculated: the 2D position of the transport component in the horizontal plane. Transport velocity was calculated as the first derivative of wrist position. Peak of transport velocity (PV) and time to peak transport velocity (TPV) were subsequently obtained. In order to account for variability in movement times between subjects and trial condition for, time to peak measures were time normalized as percentage of MT (relative time to peak aperture - rTPA, and relative time to peak transport velocity - rTPV). Finally, hand distance to the object at peak aperture - hdPA (the 2D distance from wrist marker to the object at peak aperture) was used to determine whether the lack of haptic feedback affects the organization of aperture closure.

### Statistics

Linear Pearson’s correlations were used to compare aperture and transport velocity trajectories. A 2 × 3 × 3 repeated measures analysis of variance (rmANOVA) with factors Environment (PE, VR), Object Size (Large, Medium, Small), and Object Distance (Near, Middle, Far) was used to evaluate differences between the environments, separately for all calculated kinematics.

A 2 × 3 rmANOVA with factors Environment (PE, VE) and Phase (Initiation, Shaping, Closure, see definitions in the ‘three phases of the reach-to-grasp movement’ paragraph) was utilized to track differences between environments and phases of reach-to-grasp movements, see also Fig. 7. The Shapiro-Wilk test was used to verify the normality of data distribution; all variables met this assumption. The Mauchly’s sphericity test was used to validate assumptions for repeated measures ANOVAs; Greenhouse-Geisser corrections were applied to the factors that violated the assumption of sphericity. The Levene’s test was used to verify homogeneity of the samples data variance; all data satisfied the assumption of variance homogeneity’.

Significant effects were further explored using pairwise contrasts with Bonferroni corrections.

To quantify reach-to-grasp coupling, linear Pearson correlations between TPA and TPV (both real and normalized) were calculated. To compare slopes between linear regressions and to assess the vertical distance between the thumb and index finger positions both for PE and VE t-test was used. The threshold for statistical significance was set as at *p* < .05. All statistical analyses were performed using Statistica (ver. 13, Dell Inc.). All variables are presented as means with standard deviations.

## Results

### Comparison of trajectories between physical and virtual environments

**Fig. 2:**
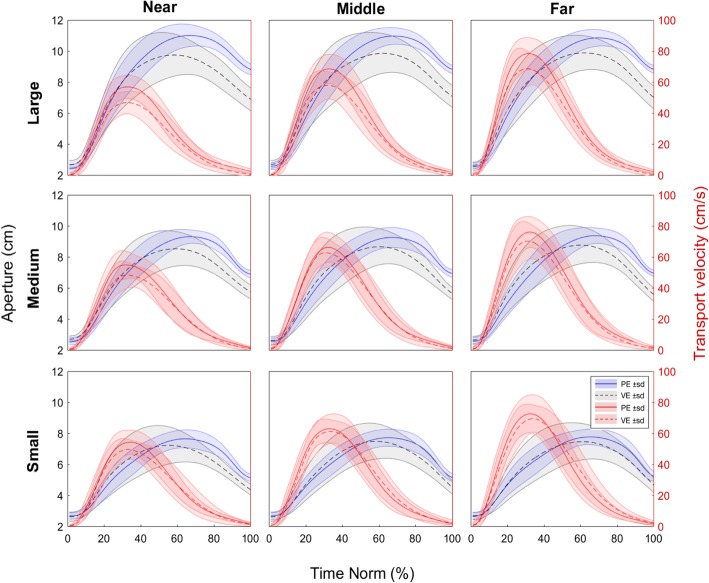
Grip aperture (left y axis, blue and grey) and transport velocity (right y axis, red) during reach-to-grasp movement for all conditions. Presented data are time normalized, profiles showed means with standard deviations, solid lines PE, dashed lines VE

**Fig. 3:**
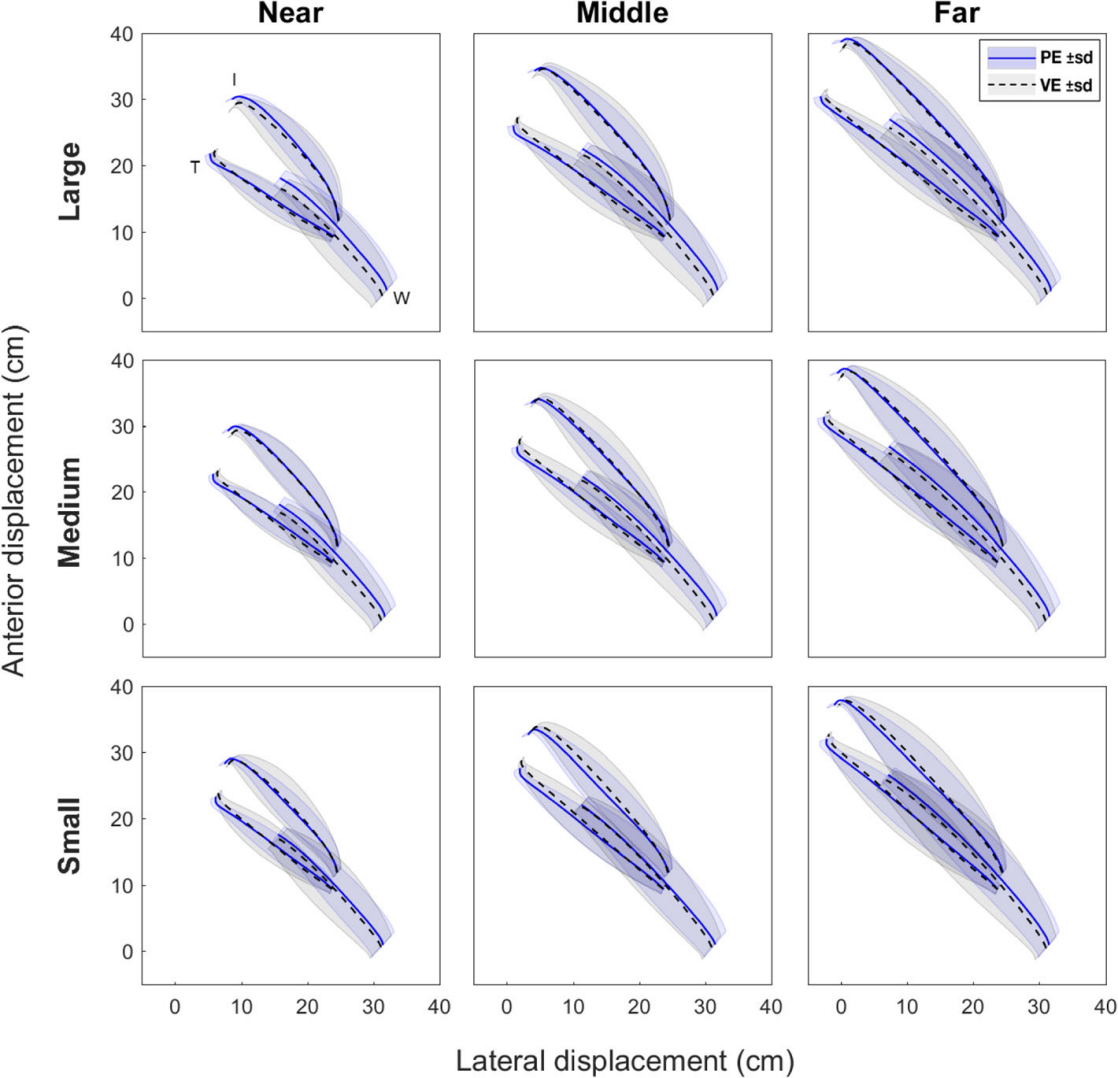
Trajectories of reaching (wrist marker - W) and grasping (index finger marker – I, and thumb marker - T) averaged across all subjects, for each condition. Solid and dashed lines represent the mean trajectories, with shaded areas representing the standard deviation

Correlational analyses examining differences in the relationship between aperture and transport velocity profiles between the physical (PE) and virtual (hf-VE) environment indicated largely invariant movement patterns (see Fig. 2). On average, across all subjects and conditions, correlation coefficients were r = 0.97 ± 0.03, *p* < .001, and r = 0.95 ± 0.05, *p* < .001 for velocity and aperture, respectively. Pearson’s correlation coefficients between all conditions are presented in Table 1. Invariant movement patterns are also evident when the positions of individual markers are compared in the horizontal plane. Figure 3 shows the mean positions of markers corresponding to the transport (wrist marker - W) and the grasp (thumb - T and index finger - I), overlaid for the two environments and for all conditions.

**Table 1:** Pearson correlation between physical and virtual transport velocity and aperture profiles

PE vs VE profiles		Transport velocity	Aperture
Large	Near	0.96 ± 0.03	0.95 ± 0.05
	Middle	0.96 ± 0.02	0.96 ± 0.05
	Far	0.97 ± 0.03	0.96 ± 0.04
Medium	Near	0.98 ± 0.02	0.96 ± 0.04
	Middle	0.96 ± 0.01	0.95 ± 0.05
	Far	0.98 ± 0.02	0.94 ± 0.04
Small	Near	0.96 ± 0.03	0.96 ± 0.07
	Middle	0.97 ± 0.03	0.96 ± 0.04
	Far	0.96 ± 0.04	0.97 ± 0.03
Average:		**0.97 ± 0.03**	**0.95 ± 0.05**

All values are significant, *p* < 0.001, PE - physical environment, VE - virtual environment, data represents mean with standard deviation

### Comparison of kinematics between physical and virtual environments

All calculated spatial and temporal kinematics (mean, sd), separately for all conditions are presented in Table 2.

**Table 2:** Kinematics calculated during reach-to-grasp movement for all conditions

Variables/Conditions		Large			Medium			Small		
		Near	Middle	Far	Near	Middle	Far	Near	Middle	Far
Movement Time MT (ms)	PE	904.3	977.0	1027.3	934.5	1002.8	1057.4	939.3	1033.3	1121.7
	sd	±95.0	±119.7	±117.2	±115.5	±112.5	±120.0	±119.6	±138.1	±167.5
	VE	1053.1	1113.8	1144.6	1001.6	1037.2	1134.8	978.1	1025.5	1080.9
	sd	±157.4	±155.6	±143.5	±170.9	±143.2	±156.5	±135.5	±125.1	±150.5
Peak Aperture PA (cm)	PE	11.1	11.1	11.0	9.5	9.4	9.6	7.8	7.9	7.9
	sd	±0.6	±0.5	±0.5	±0.5	±0.6	±0.5	±0.6	±0.5	±0.5
	VE	10.0	10.1	10.1	8.7	9.0	9.0	7.5	7.7	7.7
	sd	±1.3	±1.3	±1.1	±1.2	±1.3	±1.2	±1.2	±1.1	±1.2
Relative Time to Peak Aperture rTPA (%)	PE	66.4	67.1	69.0	66.1	67.4	66.7	64.4	65.5	65.1
	sd	±6.2	±5.4	±5.2	±5.9	±6.2	±6.6	±5.8	±6.9	±7.0
	VE	60.2	61.7	61.8	58.8	59.3	60.7	57.1	59.7	61.5
	sd	±9.1	±8.4	±9.0	±7.1	±9.1	±9.6	±9.8	±8.9	±9.0
Size Normalized Peak Aperture snPA (a.u.)	PE	1.4	1.4	1.3	1.6	1.5	1.6	2.1	2.1	2.1
	sd	±0.1	±0.1	±0.1	±0.1	±0.2	±0.2	±0.3	±0.3	±0.3
	VE	1.7	1.7	1.7	2.0	2.1	2.2	2.7	2.6	2.5
	sd	±0.3	±0.2	±0.2	±0.3	±0.3	±0.4	±0.7	±0.6	±0.7
Peak Transport Velocity PV (cm/s)	PE	61.6	73.2	83.5	60.2	69.9	82.5	57.8	68.2	77.7
	sd	±7.0	±9.1	±10.7	±8.1	±9.7	±11.1	±7.6	±9.0	±11.9
	VE	51.5	64.0	74.3	52.9	67.4	75.8	53.6	66.2	74.4
	sd	±6.3	±9.6	±13.3	±8.9	±9.6	±11.9	±6.7	±7.9	±9.5
Relative Time to Peak Transport Velocity rTPV (%)	PE	32.0	31.5	31.7	31.6	32.6	32.0	34.0	32.3	31.9
	sd	±5.4	±4.5	±4.8	±6.1	±3.6	±5.0	±4.5	±5.3	±4.4
	VE	33.2	32.7	32.0	34.3	32.0	32.5	34.8	34.6	35.0
	sd	±6.8	±6.7	±5.5	±6.1	±5.0	±5.6	±6.2	±5.4	±5.1
Hand Distance to the Object at Peak Aperture hdPA (cm)	PE	2.5	2.6	2.7	2.5	2.6	3.0	3.1	3.1	3.5
	sd	±0.7	±0.9	±0.8	±0.9	±0.9	±1.1	±1.2	±1.0	±1.4
	VE	3.3	3.6	4.2	4.0	4.3	4.8	4.6	5.0	5.7
	sd	±1.5	±1.7	±2.0	±1.6	±2.1	±2.6	±1.8	±2.2	±2.4

*PE* - Physical environment, *VE* - Virtual environment, data represents mean with standard deviation (sd)

### Movement time

A 2 × 3 × 3 repeated measures ANOVA (see Table 3) revealed a significant main effect of Environment for MT, (F_(1, 12)_ = 5.55, *p* = 0.037, η^2^ = 0.32). On average, subjects needed more time to reach-to-grasp objects in VE (1063.3 ± 40.9 ms) vs. PE (999.7 ± 32 ms). Expectedly, the analysis also showed a main effect of factor Distance (F_(1.3, 16)_ = 111.79, *p* < 0.0001, η^2^ = 0.9). Analysis of the interaction between Environment and Size factors (F_(2, 24)_ = 13.51 *p* < 0.0001, η^2^ = 0.53), showed that subjects required more time to grasp the Large object in VE (1103 ± 159.1 ms) than in PE (969.5 ± 123.7 ms), post-hoc (*p* < 0.0001, d = 0.94). The differences in MT between Medium and Small object sizes across environments were not significant, see Fig. 5, panel a. The significant interaction in MT suggests that differences between environments are size-, but not distance-dependent. Since MT between environments exhibited significant differences, further analyses of temporal features were conducted using time-normalized data.

**Table 3:** The 2 × 3 × 3 repeated measure analysis of variance ANOVA

Variables	Environment (E)	Size (S)	Distance (D)	Interaction	Figure 5
Movement Time MT (ms)	F_(1, 12)_ = 5.5 *p* = 0.037 η^2^ = 0.32	NS	F_(1.3, 16)_ = 111.8 *p* < 0.0001 η^2^ = 0.9	E*S F_(2, 24)_ = 13.5 *p* < 0.0001 η^2^ = 0.53	panel a
Peak Aperture PA (cm)	NS	F_(2, 24)_ = 697.3 *p* = 0.0001 η^2^ = 0.98	F_(2, 24)_ = 6.6 *p* = 0.0051 η^2^ = 0.35	E*S, F_(1.3, 13.5)_ = 12.8 *p* = 0.002 η^2^ = 0.52 E*D F_(2, 24)_ = 5.3 *p* = 0.012 η^2^ = 0.3	
Relative Time to Peak Aperture rTPA (%)	F_(1, 12)_ = 20.2 *p* = 0.0007 η^2^ = 0.63	F_(2, 24)_ = 3.9 *p* = 0.034 η^2^ = 0.24	F_(2, 24)_ = 17.7 *p* = 0.0001 η^2^ = 0.6	NS	
Size Normalized Peak Aperture snPA (a.u.)	F_(1, 12)_ = 24.2 *p* = 0.0003 η^2^ = 0.67	F_(1, 24)_ = 84.9 *p* = 0.0001 η^2^ = 0.88	NS	NS	panel b
Peak Transport Velocity PV (cm/s)	F_(1, 12)_ = 26.2 *p* = 0.0002 η^2^ = 0.69	NS	F_(1.25, 15)_ = 254.3 *p* = 0.0001 η^2^ = 0.95	E*S F_(2, 24)_ = 6.9 *p* = 0.0042 η^2^ = 0.37	panel c
Relative Time to Peak Transport Velocity rTPV (%)	NS	F_(2, 24)_ = 4.7 *p* = 0.018 η^2^ = 0.28	NS	NS	
Hand Distance to the Object at Peak Aperture hdPA (cm)	F_(1, 12)_ = 15.6 *p* = 0.0019 η^2^ = 0.56	F_(2, 24)_ = 20.2 *p* = 0.0001 η^2^ = 0.63	F_(1.4, 15)_ = 13.6 *p* = 0.0008 η^2^ = 0.36	E*S F_(2, 24)_ = 6.7 *p* = 0.0046 η^2^ = 0.28	panel d

*NS* – Not significant

### Peak aperture

As expected, we noted a significant main effect of PA for factor Size (F_(2, 24)_ = 697.35, *p* = 0.0001, η^2^ = 0.98), indicating that aperture was scaled to target object size. There was a significant interaction between factors Environment and Size (F_(1.3, 13.5)_ = 12.8, *p* = 0.002, η^2^ = 0.52). Post-hoc analyses showed significant differences between Large (*p* < 0.001, d = 1.01) and Medium (*p* < 0.001, d = 0.59) objects when comparing grasping in PE and VE. Interestingly, a significant main effect of Distance was also found for PA (F_(2, 24)_ = 6.61, *p* = 0.005, η^2^ = 0.35). Subsequent post-hoc analyses revealed that this effect was due to differences between grasps executed to the Near and Far distances (d = 0.08). The Environment and Distance interaction was also significant (F_(2, 24)_ = 5.26, *p* = 0.012, η^2^ = 0.3), with post-hoc tests showing significant differences between environments at all of the distances (*p* values< 0.01, d values > 0.35).

### Relative time to peak aperture

Analysis of rTPA revealed a significant main effect of Environment (F_(1, 12)_ = 20.18, *p* = 0.0007, η^2^ = 0.63), showing that peak aperture occurred later in PE compared to VE. Moreover, the Size (F_(2, 24)_ = 3.88, *p* = 0.034, η^2^ = 0.24) and Distance (F_(2, 24)_ = 17.74, *p* = 0.0001, η^2^ = 0.6) factors also showed significant differences. Post-hoc tests indicated significant differences between Large and Small objects for Size (*p* = 0.031, d = 0.25), as well as between Near and Middle (*p* = 0.002, d = 0.15), and Near and Far (*p* < 0.001) distances for the Distance effect. There were no significant interactions.

### Size normalized peak aperture

**Fig. 4:**
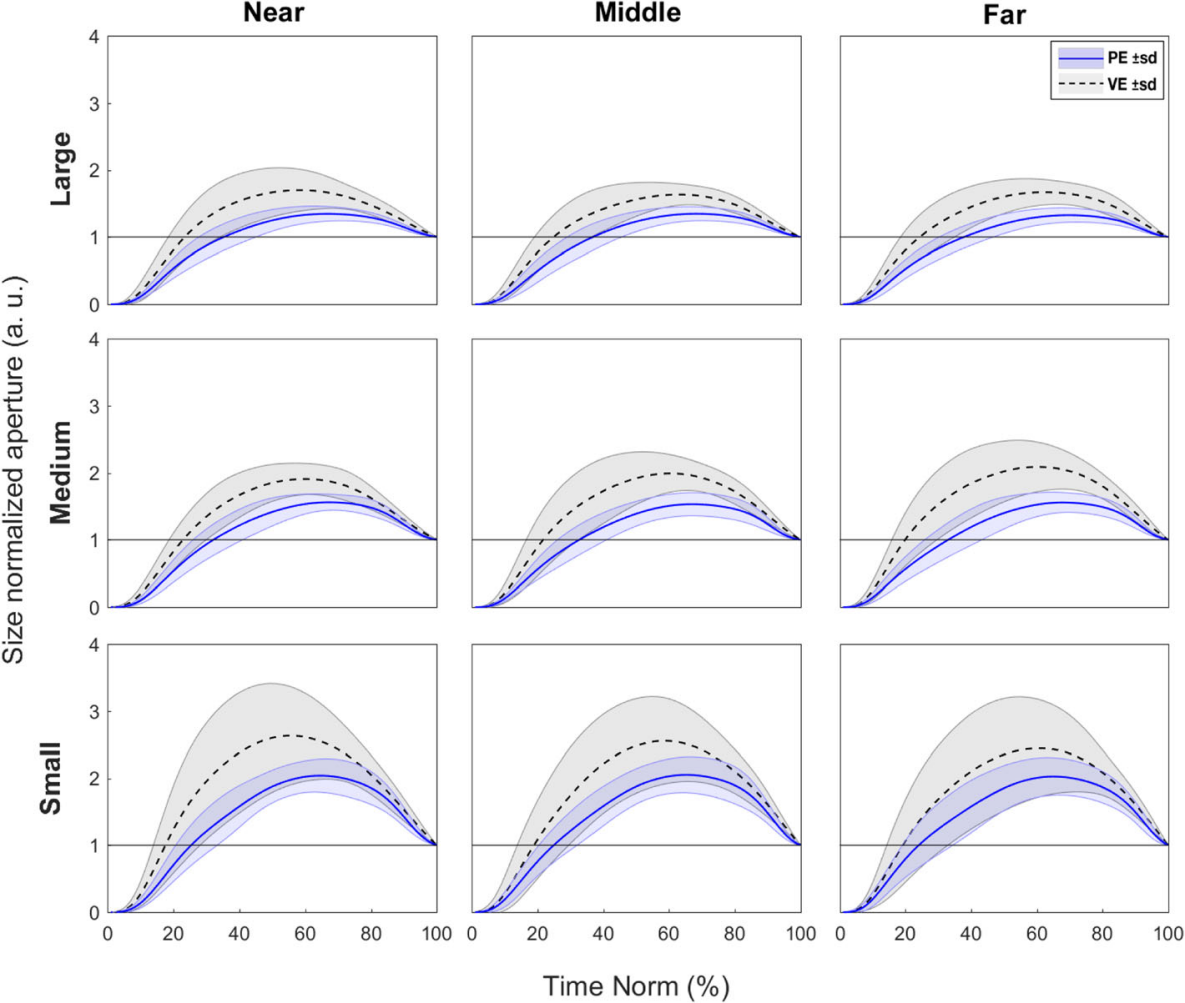
Size normalized aperture. Note that initial aperture was normalized to zero by removing the distance between the digits at movement onset, and final aperture was normalized to 1, in order to standardize grasp aperture as a proportion of opening relative to each object size (represented by the horizontal lines at y = 1)

**Fig. 5:**
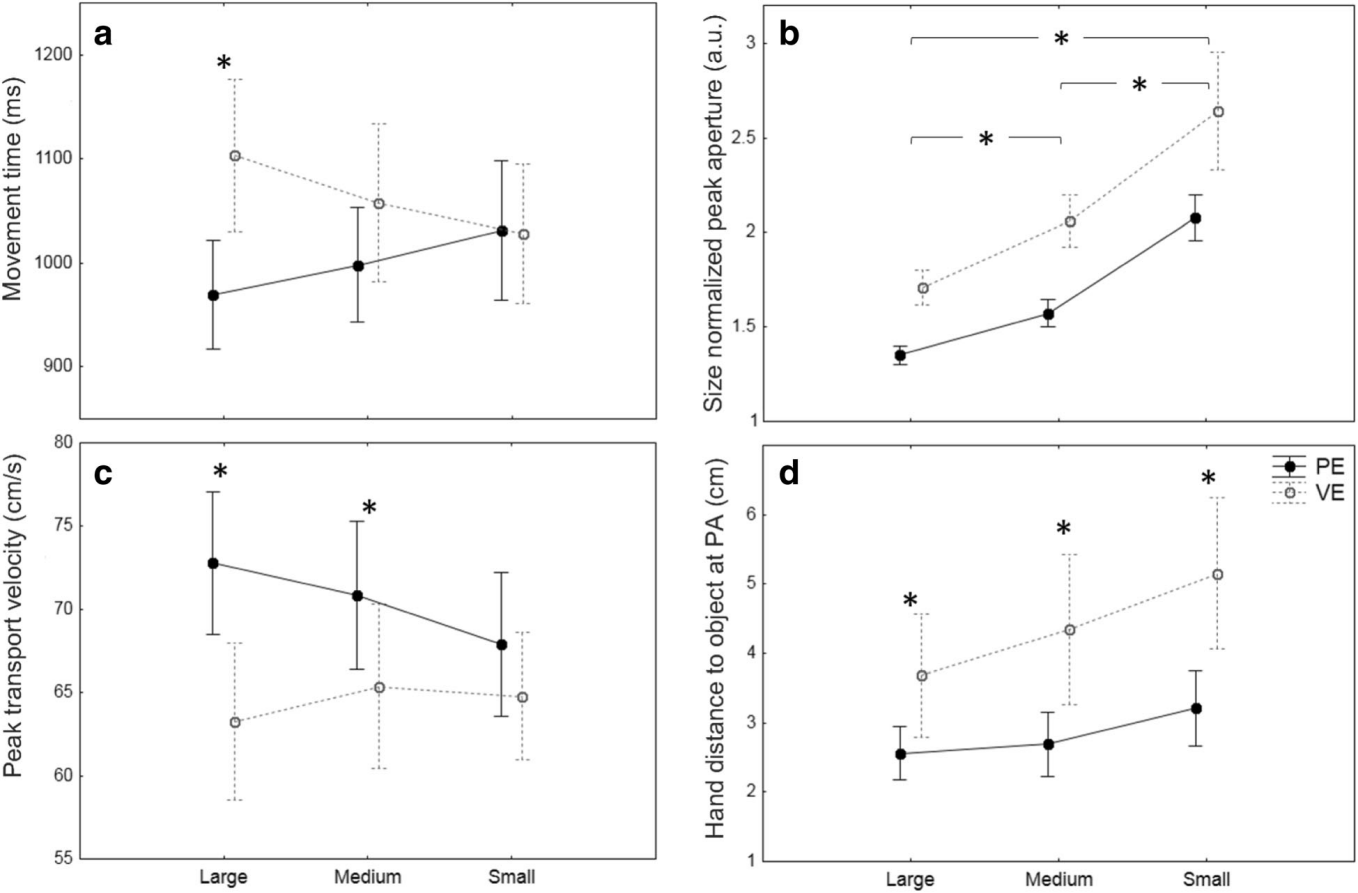
Panels **a, c, d -** significant interactions between Environment and Size factors, **a** - movement time, **c** - peak transport velocity, and **d** - hand distance to the object at peak aperture. Panel **b** - comparison of aperture overshooting between environments and object sizes. **b** - size normalized peak aperture, * - significant post-hoc between PE and VE conditions, data represents means with standard deviations

In order to take a closer look at the effect on PA between environments regardless of object size, we performed a rmANOVA on the PA measures normalized to object size. Analysis revealed a significant main effect of Environment (F_(1, 12)_ = 24.2, *p* = 0.0003, η^2^ = 0.67) indicating greater overshoot when grasping the virtual objects. Post-hoc analyses showed significant differences between all object sizes (*p* values < 0.001, d values > 1.09), indicating that participants opened their hand proportionally wider for smaller objects (the ‘margin of safety’ was not the same for all object sizes), see Fig. 5, panel b. Note that variability was also larger in VE, which might reflect uncertainty of grasping the virtual objects (see Fig. 4). There were no other significant main effects nor interactions.

### Peak transport velocity

There was a significant main effect of Environment for PV, (F_(1, 12)_ = 26.24, *p* = 0.0002, η^2^ = 0.69), which confirms the fact that movement speed (on averages 70.5 ± 2.35 cm/s for PE vs 64.5 2.58 cm/s in VR) was faster in the physical relative to the virtual environment. As expected, no main effect of Size was found (F_(1, 12)_ = 3.1, *p* = 0.068, η^2^ = 0.2), however a significant main effect of Distance (F_(1.25, 15)_ = 254.3, *p* = 0.0001, η^2^ = 0.95), indicated that the further the object was from the starting position, the faster the transport component. Similar to MT, PV showed a significant =interaction between Environment and Size (F_(2, 24)_ = 6.93, *p* = 0.0042, η^2^ = 0.37). Post-hoc tests revealed significant differences between PE and VE for the Large (*p* < 0.001, d = 0.71) and Medium sized objects (*p* < 0.01, d = 0.4).

### Relative time to peak transport velocity

Analysis of rTPV revealed a significant main effect of Size (F_(2, 24)_ = 4.72, *p* = 0.018, η^2^ = 0.28). Post-hoc tests showed that rTPV occurred later for smaller compared to large objects (*p* = 0.023, d = 0.29). There were no other significant main effects nor interactions.

### Hand distance to the object at peak aperture

The ANOVA on hdPA showed a significant main effect of factor Environment (F_(1, 12)_ = 15.63, *p* = 0.0019, η^2^ = 0.56). In general, subjects began closing their grip relatively earlier in VE (4.4 ± 0.57 cm) compared to PE (2.8, ±0.26 cm). A significant main effect of factor Distance (F_(1.4, 15)_ = 13.65, *p* = 0.0008, η^2^ = 0.36) showed that the further the object is, the later subjects began to close their grip aperture. A significant main effect of factor Size (F_(2, 24)_ = 20.25, *p* = 0.0001, η^2^ = 0.63) revealed a similar trend, showing that the smaller the object is, the later closure of grip aperture is started. The significant interaction of factors Environment and Size (F_(2, 24)_ = 6.7, *p* = 0.0046, η^2^ = 0.28) showed that the difference of hdPA between environments is mainly size dependent. Post-hoc analyses revealed that hdPA for all object sizes was significantly different between PE and VE (*p* values < 0.001, d values > 0.75), see Fig. 5, panel d.

### Coupling between kinematics in physical and virtual environments

**Fig. 6:**
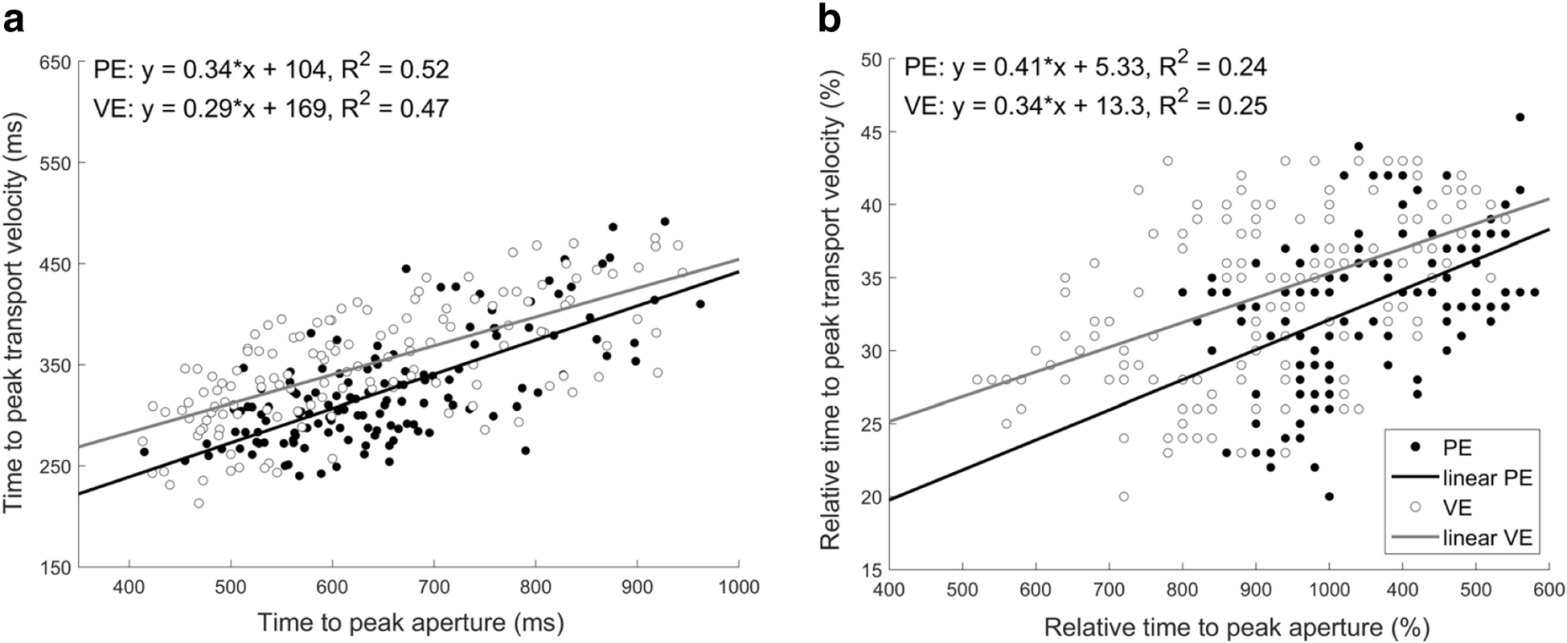
Panel **a** - linear regressions between times to peak aperture and peak transport velocity in physical (PE) and virtual environments (VE). Panel **b** - linear regressions between relative times to peak aperture and peak transport velocity in PE and VE

A graphic representation of reach-to-grasp coupling is presented on Fig. 6. First, temporal data were plotted of the reaching component against the grasping component both for PE and hf-VE. Then each of these data sets were fitted with linear regression models. All regressions were found to be significant (p values < 0.0001). Non-significant differences between PE and hf-VE in the slopes of linear fits for TPA and TPV (t = 0.16, *p* = 0.872, d = 0.25), and rTPA and rTPV (t = - 1.12, *p* = 0.285, d = 0.35), indicated that the coupling between reaching and grasping was preserved (e.g., fitted regression lines are close to parallel) and independent of the environment in which movement was performed.

### Three phases of the reach-to-grasp movement

**Fig. 7:**
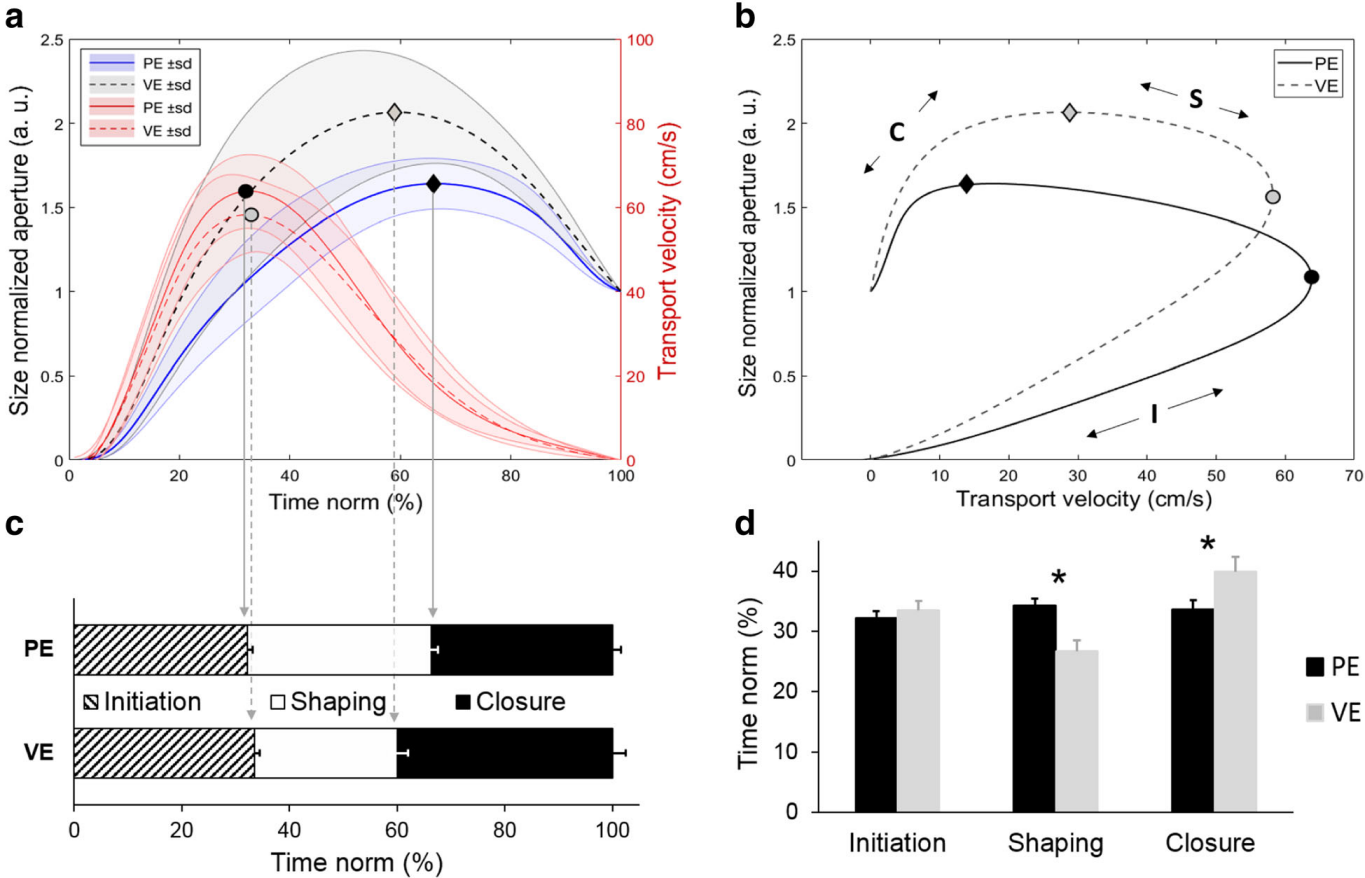
Relative time of three phases of reach-to-grasp movement averaged across all conditions. Panel **a** - grip aperture and transport velocity profiles with peak values. Panel **b** - three phases of reach to grasp movement: I - initiation phase, S - shaping phase, **c** - closure phase (described in the text); Panel **c** - relative time of individual phases between physical (PE) and virtual (VE) environments. Panel **d -** differences of individual phases between PE and VE, * - significant differences, *p* < .01

Based on the observed significant main effect and interaction between environment and object size on movement time (see Fig. 5, panel a), we decided it would be interesting to further investigate differences in movement time at a more granular level. To do so, we plotted transport velocity against size-normalized aperture. The resulting curves revealed three distinct phases of the reach-to-grasp movement which we labeled: **Initiation** (I) from movement onset to peak transport velocity, **Shaping** (S) from peak transport velocity to peak aperture, and **Closure** (C) from peak aperture to movement offset (see Fig. 7, panel b).

A 2 × 3 rmANOVA with factors Environment (PE, VE) and Phase (Initiation, Shaping, Closure) on the proportion of movement time accounted for by each phase, showed a significant interaction between Environment and Phase (F_(2, 24)_ = 20.5, *p* = 0.001, η^2^ = 63). Post-hoc tests revealed that the proportion of movement dedicated to the initiation phase remained the same across environments. However, there was a significant difference in the proportion of the total movement time during the shaping phase (*p* = 0.001, d = 1.32), with the time allotted in VE reduced, possibly in order to enable more precise grasping during the closure phase (*p* = 0.006, d = 0.85), (see Fig. 7, panel c and d).

### Digits position

**Fig. 8:**
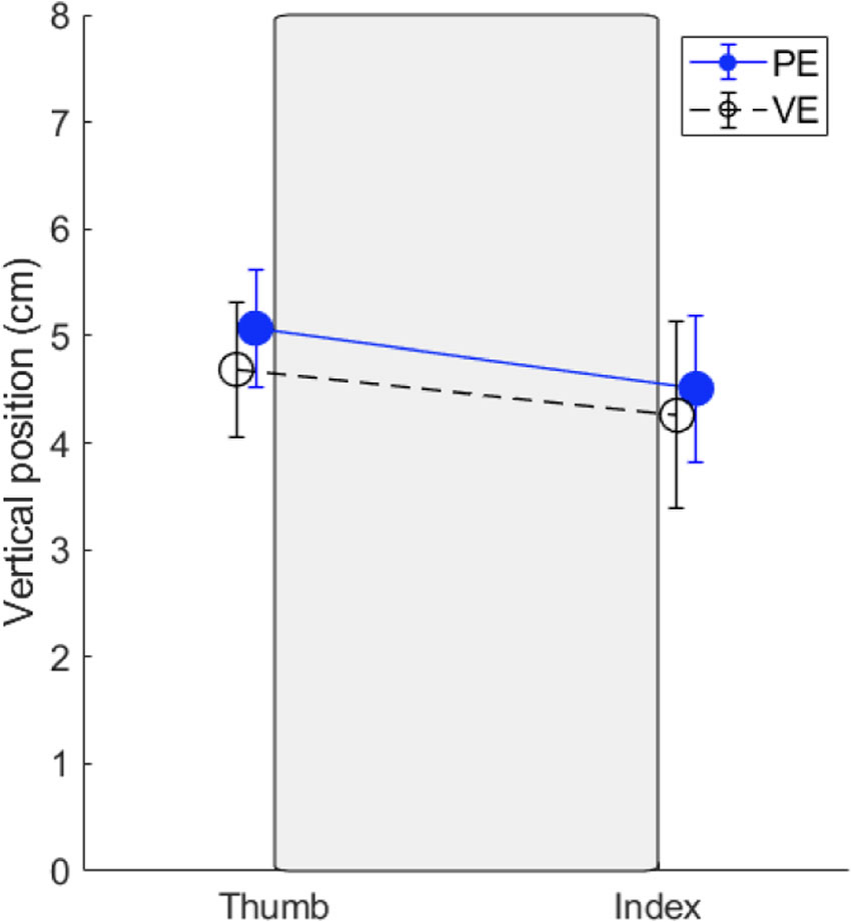
Digit positions (thumb and index fingertips) along the vertical edges of the object (gray area) at the moment of object grasp averaged across all participants and conditions, PE – physical environment, VE – virtual environment

The analysis of vertical distance between the thumb and index at movement offset (object grasp) showed no significant differences between physical (0.56 ± 0.58), and virtual (0.42 ± 0.53) environment (t = 1.81, *p* = 0.07, d = 0.25), see Fig. 8.

## Discussion

The main goal of this study was to determine the potential of current VR systems as a platform for research and rehabilitation [4, 11, 24]. In order to assess the characteristics of movements produced in VR, we tested healthy participants in a comparable reach-to-grasp task in both the PE and the VE. Importantly, we decided not to include haptic feedback, as previous studies have, due to the relatively lower state of development of that technology which results in reduced accessibility and practicality [1, 8, 9].

While most of the previous work involving VE has provided haptic feedback to the participants [13, 14, 25], we were interested in determining whether visual feedback alone would support the production of properly coordinated reach-to-grasp movements. The characteristics of prehension movements observed in our study showed that even when haptic information is absent during reach-to-grasp actions in VE, the behavior across environments showed very similar kinematic patterns. High correlations between aperture (r = 0.95 ± 0.05) and transport velocity profiles (r = 0.97 ± 0.03, see Fig. 2), as well as similar trajectories of individual markers (index finger, thumb and wrist, see Fig. 3) support this notion. Similarly, as expected, grasp aperture was scaled to object size independently of the environment where the movement was carried out. These results coincide with previous studies comparing movement kinematics in PE and VE [13, 14, 26].

Many studies have postulated that in order to grasp any object successfully, the transport and grasp components must be coordinated [17, 18, 27, 28]. Moreover, the temporal relationship between the components appears to depend on task goal, object properties, and experience [18]. In our setup we observed that even if there were some differences in movement kinematics, coordination between the reach and grasp components was preserved across environments (see Fig. 6). Similarly, though the typical landmarks of the movement were slightly different across environments, in VE they still occurred within normal ranges: TPA occurred between 60 and 70%, and TPV between 30 and 40% of movement time [16, 17, 19]. We suggest that coordination across environments is probably a more relevant measure of whether VE is useful for research and rehabilitation than any specific landmark. The fact that people produce natural movements (they do not move ‘robotically’) suggests that they are selecting and deploying the appropriate movements at relatively consistent latencies.

Having said that, the differences we observed between environments as the participants deployed their grasping movements are important to understand for the possible improvement of this technology. For example, MT exhibited differences between PE and VE, as did hdPA and snPA. Some of these results were not unexpected, as previous studies have found that in VE individuals move slower, particularly showing longer deceleration times both during reaching and grasping as well as in pointing tasks [13, 14, 25, 26, 29]. Similarly, the increase in peak aperture we observed in VE for small-sized objects, was previously reported by [13]. Larger grip aperture in VE (especially during grasps to smaller objects) have been reported in previous studies comparing physical and virtual environments [13, 14]. The effects described suggest to us there was increased perceptual uncertainty during target acquisition in VE. Therefore, a strategy to increase the safety margin in terms of visibility of both the target and the hand during the movement is needed [30]. Given the geometry of our setup, where the marker (avatar) representing the tip of the index finger is eclipsed by the distant edge of the virtual object near the time of peak aperture, it is possible that participants required more time and larger distance between the digits compared to PE, when acquiring the target object. This effect was evident when the large virtual object was reached and grasped. The analysis of the timing of the reach-to-grasp phases confirmed that subjects needed noticeably more time during the closure phase in VE (see Fig. 7).

The demarcations of the reach-to-grasp phases (shown in Fig. 7) could be useful for researchers and clinicians as a means of evaluating the proportion of total movement time spent in each phase of the reach-to-grasp movement. This analysis differentiates between a mostly internally guided - initiation phase, an execution phase when sensory feedback of object state is obtained, and a closure phase when handshape is adjusted. Based on the proportion of reach-to-grasp phases, the programming and evaluation of functional recovery process could be better understood and tailored to individual patient needs.

A related question involves the participants’ behavioral response to the collision detection algorithm employed in our study. The collision detection algorithm determines whether the limits of a virtual object collide with those of the thumb and index finger avatars [31–33]. Depending on the dimensions of the overlap allowed by the algorithm, an object may be too hard or too easy to ‘contact’. Therefore, this algorithm must be carefully calibrated in order to adjust collision detection to the participant’s ability. In this experiment, the virtual objects changed color once collision with the avatars was detected, allowing the subject to grasp and lift the object [34]. Therefore, as participants gauged the appropriate digit location to trigger the grasp they seemed to have taken longer to close their digits. This effect was observed as a relatively early beginning of aperture closing in VE (hand distance to object at peak aperture). We believe that it is possible to control a large portion of such uncertainty by modifying the collision detection algorithm and we are currently testing this possibility.

Finally, it is important to discuss the possibility that adding haptic feedback to virtual reality environments may result in a reduction of the perceptual uncertainty our participants encountered [35]. It is possible that, in the absence of haptic feedback, grasping an object became an aperture matching task under visual guidance, which might be a limitation of the study. While that may be the case, our participants were able to produce natural goal-directed movements within hf-VE.

Furthermore, lack of differences in the vertical distance between the digits at object grasp indicates that participants treated the virtual object as if it had physical properties. Placement of the digits at object contact in order to prevent roll of the object during grasp and lift has been previous shown to indicate anticipatory force control [36]. In the VE utilized here, nothing prevented the participants from grasping the virtual object with misalignment of the thumb and index, which would cause a rotation of a real object. However, this was not observed and, on average, the vertical distance between the thumb and index was in fact smaller in VE than PE. Cumulatively these results suggesting that visual feedback in current high-end virtual reality systems may be sufficient for research and rehabilitation purposes if tasks are carefully designed and calibrated.

## Conclusions

We found that prehension movements are largely similarly coordinated in both physical and our 3D-immersed virtual environments. That is, the intrinsic plans for reach-to-grasp in hf-VE did not change movement structure nor the motor programs deployed. Nonetheless, we observed that intrinsic motor plans in VE may be tuned (scaled), particularly in the closure phase, perhaps due to perceptual uncertainty and/or due to lack of haptic feedback. Therefore, we suggest that if the collision detection algorithm is properly controlled, VR may be suitable for accurate and precise reach-to-grasp movements since the virtual environment presents appropriate visuo-motor scaling for reach-to-grasp coordinated action towards different object sizes and distances. This property, as well as the relatively easy manipulation of visual feedback, leverages VR as a flexible tool for research and rehabilitation.

## Abbreviations

C: closure phase
hdPA: hand distance to the object at peak aperture (cm)
hf-VE: haptic free virtual environment
HMD: head-mounted display
I: initiation phase
MT: movement time (ms)
PA: peak aperture (cm)
PE: physical environment
PV: peak transport velocity (cm/s)
rTPA: relative time to peak aperture (%)
rTPV: relative time to peak transport velocity (%)
S: shaping phase
snPA: size normalized peak aperture (a.u.)
VE: virtual environment
VR: virtual reality
